# Biological and Health-Promoting Potential of Fruits from Three Cold-Hardy Actinidia Species

**DOI:** 10.3390/molecules30020246

**Published:** 2025-01-09

**Authors:** Piotr Latocha, Ana Margarida Silva, Manuela M. Moreira, Cristina Delerue-Matos, Francisca Rodrigues

**Affiliations:** 1 Department of Environmental Protection and Dendrology, Institute of Horticultural Sciences, Warsaw University of Life Sciences–WULS–SGGW, 02-776 Warsaw, Poland; 2 REQUIMTE/LAQV, ISEP, Polytechnic of Porto, Rua Dr. António Bernardino de Almeida, 431, 4200-072 Porto, Portugal; ana.silva@graq.isep.ipp.pt (A.M.S.); manuela.moreira@graq.isep.ipp.pt (M.M.M.); cmm@isep.ipp.pt (C.D.-M.)

**Keywords:** kiwiberry, silver vine, antioxidant activity, phenolics, Caco-2, HT29-MTX

## Abstract

Fruits are essential components of the human diet, valued for their diverse bioactive compounds with potential health-promoting properties. This study focuses on three cold-hardy *Actinidia* species, namely *A. arguta*, *A. kolomikta*, and *A. polygama*, examining their polyphenolic content, antioxidant/antiradical activities, scavenging capacity and effects on intestinal cell viability (Caco-2 and HT29-MTX). A comprehensive profile of their phenolic compounds was identified, in descending order of total polyphenol content: *A. kolomikta* > *A. arguta* > *A. polygama*. Across species, 16 phenolic acids, 2 flavanols, 2 flavanones, 11 flavonols, and 3 flavones were quantified, with caffeine as a prominent compound. *A. kolomikta* achieved the highest antioxidant activity, with ‘Vitakola’ cultivar showing almost double the antioxidant activity compared to ‘Tallinn’ and ‘Pozdni’. By contrast, *A. arguta* ‘Geneva’ and *A. polygama* ‘Pomarancheva’ exhibited significantly lower activity in both FRAP and DPPH assays. Notably, *A. kolomikta* cultivars showed distinct radical-scavenging capacities, particularly for superoxide, wherein ‘Tallinn’ and ‘Pozdni’ achieved the highest values. Cell viability tests on Caco-2 and HT29-MTX cells revealed a dose-dependent reduction in viability, notably stronger in Caco-2 cells. Overall, this study underscores the therapeutic potential of *Actinidia* species.

## 1. Introduction

Recently, there has been growing interest in the bioactive compounds found in fruits and their beneficial effects on human health. They are seen as a panacea for consuming heavily processed foods, which are known to be responsible for generating numerous reactive free radicals causing health hazards to humans, widely considered to be the cause of many civilizational diseases. Berries are classified as a rich source of bioactive compounds [1,2]. Among them, the fruits of various species of Actinidia (*Actinidia* spp.) stand out. Although the genus *Actinidia* is considered a rich source of bioactive compounds with significant health-promoting properties, there are large differences between species [3,4] or within species [5,6], irrespective of the cultivation conditions [7]. The most popular and widely cultivated species of *Actinidia* in warm climates is *A. chinensis*. Its nutrient value and health properties are fairly well known [4]. Nonetheless, knowledge about the value of other species, especially those with smaller fruits that can grow in colder climates, is scarce. Many of them have been used in folk medicine for centuries to combat different diseases [8] due to their antioxidant, antimicrobial, anti-inflammatory, antidiabetic, antiproliferative, anti-angiogenic, anticholinergic, anti-tumoral, and anticancer activities [8,9].

Among the cold-hardy kiwifruit, the most important are currently *A. arguta*, *A. kolomikta* and *A. polygama*. It seems that *A. arguta* has the greatest potential of these species and is cultivated commercially in many countries, being introduced to the market as kiwiberry or minikiwi. Its cultivation area is systematically increasing but, due to easily perishable properties and poor transportation, the fruit is mainly available at local markets. Early and recent research has pointed out *A. arguta* fruit as not only having strong antioxidant potential [7] but also showing promise in the prevention of various chronic diseases, such as hypercholesterolemia [10], some types of cancer [11,12] and neurodegenerative diseases such as Alzheimer’s or Parkinson’s [13]. *A. kolomikta* is cultivated in countries with a climate that is too cold for *A. arguta*, such as Lithuania, and are a rich source of polyphenols [14]. *A. polygama* is a species with great potential, and it has been used mainly in Chinese and Korean folk medicine to treat pain, gout, and inflammation [15]. Some researchers reported α-glucosidase inhibitory activity and insulin secretion effects in *A. polygama* fruit [16]. Actinidia fresh and processed fruits, or even waste from their production, are of great nutritional value [17].

In light of the available research, *A. arguta* is considered one of the most nutritious fruits, containing over 20 groups of various chemical compounds, including vitamins (C, B-complex, and E), carotenoids, and phenolic compounds [18,19]. In their publication, Baranowska and Wójcik [17] report that *A. arguta* fruits may contain up to 214.8 mg gallic acid equivalent (GAE)/100 g fresh weight (FW) of total polyphenols, while other authors suggest that they contain over 360 mg GA/100 g FW [20]. Among the polyphenols in *A. arguta*, 22 chemical compounds were identified, including flavonoids and organic acids [19] and, according to Baranowska and Wójcik, also anthocyanins [18]. *A. kolomikta* contains a similar chemical composition but in different proportions [6,14]. According to Zuo et al. [20], *A. kolomikta* may contain up to 430 mg/100 g FW of total polyphenols and, according to Cesoniene [14], from 720 to 1684 µg/g dry weight depending on the cultivar. In the fruits, 14 compounds classified as flavan-3-ols, flavones, phenolic acids, and flavonols have been identified. On the other hand, other studies [21] indicate that *A. kolomikta* fruits containing 7.75–10.25 mg GAE/g FW are almost 10 times richer in phenolic compounds than *A. polygama* fruit, in which the authors determined only 0.56–1.19 mg GAE/g FW. Another study conducted on *A. polygama* [22] showed similar relationships, where both the peel and pulp of *A. arguta* fruit (374 and 175.2 mg GAE/100g FW, respectively) were richer sources of polyphenols than the peel and pulp of *A. polygama* (291.9 and 136.2 mg GAE/100g FW, respectively). Relatively least known is the chemical composition of *A. polygama* [16,23].

In recent years, there has been more information presented about the anti-cancer and anti-degenerative properties of *Actinidia* species, in not only fresh fruits but also processed ones [12,13], leading to an increased interest in these species as a source of fresh and processed fruits (e.g., juices or dietary supplements). However, to the best of our knowledge, no comprehensive comparative studies of these species have been conducted on fruits from plants grown in analogous conditions. This allows eliminating environmental factors and focusing only on genetic differences. Therefore, the present study intends to compare the antioxidant/antiradical activity, radical scavenging capacity, phenolic profile, and antitumor potential in intestinal cells of extracts from fruits of three species and several cultivars of *Actinidia* grown in the same climatic and habitat conditions, namely *A. arguta, A. polygama* and *A. kolomikta*.

## 2. Results and Discussion

### 2.1. Total Phenolics Content (TPC), Antioxidant Activities and Phytochemical Profiles

Table 1 summarizes the different samples’ TPC and antioxidant/antiradical activities. Regarding TPC and the total sum of identified phenolic compounds, the genotypes studied can be arranged in the following order: *A. kolomikta* ‘Vitakola’ > *A. kolomikta* ‘Tallinn’ > *A. kolomikta* ‘Pozdni’ > *A. arguta* ‘Geneva’ > *A. polygama* ‘Pomarancheva’. A significantly higher TPC content was found in the extracts from *A. kolomikta* fruits, although significant differences are visible within the studied cultivars. The highest TPC content was measured in the ‘Vitakola’ cultivar (110.05 mg GAE/g dry weight (dw), which is 9.4-fold and 13.2-fold higher than the contents found in *A. arguta* ‘Geneva’ and *A. polygama* ‘Pomarancheva’, respectively. Slightly less was identified in the other two cultivars.

Qualitative analysis of the phenolic compounds allowed for the identification of 16 phenolic acids, 2 flavanols, 2 flavanones, 11 flavonols, 3 flavones and 6 other compounds, among which caffeine was dominant (Table 2). Figure S1 (Supplementary Materials) presents chromatograms of the standard solutions containing all 40 phenolic compounds, as well as an example for the analyzed sample. The dominant group of phenolic compounds were catechins from the flavanol group. The largest amount of them was contained in *A. kolomikta* ‘Vitakola’ cv. fruits (7040.5 mg/100 g dw), with slightly less in ‘Tallin’ and ‘Pozdni’ cvs. (5077.4 and 5083.1 mg/100 g dw, respectively). The least amount of these compounds, only 0.25% of that found in *A. kolomikta* ‘Vitakola’, was contained in *A. polygama* ‘Pomarancheva’ fruits (17.6 mg/100 g dw). The second dominant group of compounds were phenolic acids (Table 2). In *A. kolomikta* fruits, caftaric acid, 4-*O*-caffeoylquinic acid, and gallic acid dominated (385.9–696.9, 143.8–252.4 and 78.2–222.9 mg/100 g dw, respectively). In *A. polygama*, in addition to 4-*O*-caffeyolquinic acid, chlorogenic acid was also present at the highest concentration (79.1 g/100 g dw). In turn, in *A. arguta* ‘Geneva’ fruits, the most abundant were protocatechuic and neochlorogenic acids (177.8 and 164.1 mg/100 g dw, respectively). Apart from these dominant compounds, flavonols (89.4, 48.2–72.6 and 3.1 g/100 g dw, respectively) and flavanones (10.1, 3.9–7.7 and 1.9 g/100 g dw, respectively) were also determined in smaller amounts in *A. polygama* ‘Pomarancheva’, *A. kolomikta* and *A. arguta* ‘Geneva’ fruits. *A. arguta* ‘Geneva’ fruits were also distinguished by the content of flavones (1.2–4.7 mg/100 g dw), which were not found in the fruits of the other two species. Among other compounds, caffeine was determined in significant amounts in *A. kolomikta* and *A. arguta* fruits (46.3–63.3 and 28.5 g/100 g dw, respectively) and phloridzin in *A. polygama* fruits (27.8 g/100 g dw) (Table 2).

The obtained results show significant diversity among the species studied regarding the total content and composition of phenolic compounds. In general, *A. kolomikta* is the richest source of phenolic compounds, with a dominant share of flavanols and phenolic acids. *A. arguta* and *A. polygama* contain significantly fewer polyphenols, with dominant shares of phenolic acids and flavanols and phenolic acids and flavonols, respectively. Similar results for *A. kolomikta* and *A. arguta* were found in Česoniene et al.’s study [24] in which the content of polyphenols in *A. kolomikta* (177.80 mg/g dw) was, on average, three times higher than in *A. arguta* fruits (54.45 mg/g dw). The authors identified four phenolic acids, eight flavonols, two flavones, and five flavon-3-ols in *A. kolomikta* and *A. arguta* berry extracts. In turn, comparative studies of *A. kolomikta* and *A. polygama* conducted by Panischeva et al. [21] reported that *A. kolomikta* fruits exceeded *A. polygama* fruits 10-fold in terms of TPC. Similar studies were conducted by Khromykh et al. [22] for *A. polygama* and *A. arguta* in Ukraine. The authors showed that *A. arguta* pulp and peel are a richer source of TPC and Free Phenolic Acid Content (1.3× and 1.1×, respectively) when compared to *A. polygama* but a worse source of TFC (1.67×). The peel was a richer source of these compounds in each case than the pulp.

The strongest antioxidant activity measured by the FRAP method was found in extracts from *A. kolomikta* fruits, with the activity in *A. kolomikta* ‘Vitakola’ (1049.30 μmol FSE/g dw) being almost 2× higher than in the other two cultivars (*A. kolomikta* ‘Tallinn’ and *A. kolomikta* ‘Pozdni’, 576.24 and 517.96 μmol FSE/g dw, respectively) (Table 1). Moreover, the activity measured by this method for *A. arguta* ‘Geneva’ and *A. polygama* ‘Pomarancheva’ was over 4× lower than for *A. kolomikta* ‘Tallinn’ and *A. kolomikta* ‘Pozdni’ and over 7× lower than for *A. kolomikta* ‘Vitakola’. Regarding the DPPH assay, it was not possible to determine the IC_50_ for the *A. polygama* ‘Pomarancheva’ and *A. arguta* ‘Geneva’ extracts at the highest concentration tested (1000 µg/mL), which presented inhibition percentages of 14.20% and 17.36%, respectively.

The superoxide anion radical (O_2_^•−^) and the hypochlorous acid (HOCl) scavenging capacities are summarized in Table 1. Concerning HOCl radicals, the highest values were obtained in *A. arguta* ‘Geneva’ and *A. polygama* ‘Pomarancheva’ (51.81 and 45.61 μg/mL, respectively). At the same time, the intermediate results were achieved for *A. kolomikta* ‘Vitakola’ (11.04 μg/mL) and the lowest for *A. kolomikta* ‘Pozdni’ and *A. kolomikta* ‘Tallinn’ (9.35 and 6.37 μg/mL, respectively). No activity was observed for O_2_^•−^ scavenging capacity in *A. arguta* ‘Geneva’ and *A. polygama* ‘Pomarancheva’, even at the maximum tested concentration. The highest values were measured in *A. kolomikta* ‘Tallinn’ and *A. kolomikta* ‘Pozdni’ (102.73 and 136.61 μg/mL, respectively), while *A. kolomikta* ‘Vitakola’ (47.37 μg/mL) achieved the lowest one. To the best of our knowledge, this is the first time that these species were evaluated, so further studies on different cultivars are needed to confirm the relationships we obtained.

The analysis of correlations between TPC and individual groups of phenolic compounds conducted in the present study showed a strong correlation between TPC and Flavanols (*R*^2^ = 1.00, *p* < 0.001), others (*R*^2^ = 0.80, *p* < 0.01) and the sum of all identified compounds (*R*^2^ = 0.99, *p* < 0.001). A slightly smaller correlation was found in relation to the sum of phenolic acids (*R*^2^ = 0.76, *p* < 0.05) (Table 3). Such correlations are probably related to the quantitative share of individual groups of compounds in the total profile from the extract of individual fruits. Early and recent research has pointed out *A. arguta* fruit as promising in the prevention of various chronic diseases, i.e., cardiovascular and digestive disorders, with proven anti-allergic [25], anticholinergic, antiglycemic [9], anti-inflammatory, anti-hypercholesterolemic [10], antioxidant and hepatoprotective activities, showing a protective effect against some types of cancer [12,20,26] or neurodegenerative diseases, i.e., Alzheimer’s or Parkinson’s [13].

A correlation analysis was performed between the different assays (Table 3).

As can be observed, it is possible to conclude that there is a strong correlation between TPC and the flavanols group and the sum of all determined phenolic compounds (*R*^2^ = 0.99, *p* < 0.001). Similarly, strongly correlated with flavanols and the sum of all determined polyphenols were the antioxidant activity determined by the FRAP method (*R*^2^ = 0.90, *p* < 0.05 and 0.88, *p* < 0.05, respectively) and by HOCl (*R*^2^ = 0.89, *p* < 0.05 and 0.91, *p* < 0.05, respectively) (Table 3). Among the most strongly correlated group of polyphenols, Catechin dominated, which in *A. kolomikta* ‘Tallinn’, *A. kolomikta* ‘Vitakola’ and *A. kolomikta* ‘Pozdni’ constituted 76.2%, 85.4% and 83.5%, respectively, of all determined polyphenols. In comparison, in *A. arguta* ‘Geneva’ and *A. polygama* ‘Pomarancheva’ it constituted only 27.2% and 2.2%, respectively (Table 2). The correlation between antioxidant activity measured by the DPPH and O_2_^•−^ methods was different. Here, the effect of Flavones was the strongest (*R*^2^ = 0.80, *p* < 0.05 and 0.91, *p* < 0.05, respectively) (Table 2). Moreover, a strong correlation was found between TPC and antioxidant activity measured by the FRAP and HOCl methods (*R*^2^ = 0.94, *p* < 0.01 and 0.84, *p* < 0.05, respectively) and between DPPH and HOCl and O_2_^•−^ (*R*^2^ = 0.93, *p* < 0.01) (Table 3). These results confirm the diverse analytical mechanisms of individual methods for determining antioxidant activity, confirming the necessity of using different methods to determine the total antioxidant potential. Similar relationships between the content of polyphenols and vitamin C and antioxidant activity (*R*^2^ above 0.92 and 0.88, respectively, *p* < 0.01) measured by the PSC method (Rapid Peroxyl Radical Scavenging Capacity Assay) in *A. arguta* extracts were obtained by Zhang et al. [27], indicating that polyphenols and vitamin C significantly contributed to the in vitro antioxidant activity. Similar relationships between antioxidant activity measured by DPPH and FRAP methods and polyphenol content were found in many other fruits [28].

### 2.2. Cell Viability Assays

Caco-2 and HT29-MTX cells are commonly used as standard models for studying extracts’ effects on the human intestine [29]. Table 4 and Table 5 summarize the obtained results after exposure of HT29-MTX and Caco-2 cells to concentrations of the different extracts, respectively.

*A. kolomikta* ‘Tallinn’ and *A. kolomikta* ‘Vitakola’ led to a cell viability decrease with increasing concentration in HT29-MTX cells, while for the other genotypes, the viability did not differ significantly (Table 4). Regarding Caco-2 cells, significantly higher viability was found after exposure to the lowest concentration of *A. polygama* ‘Pomarancheva’, *A*. *kolomikta* ‘Vitakola’, and *A. kolomikta* ‘Pozdni’. Regarding *A. arguta* ‘Geneva’ and *A. kolomikta* ‘Tallinn’, a significant decrease in cell viability was noted only at the highest tested concentration (Table 5). These results align with Yu et al. [26], who identified the AAP-3 polysaccharide as responsible for the antitumor effect of *A. arguta* extract. According to the authors, the anti-proliferative effect of this polysaccharide was associated with cell cycle block and apoptosis on Hep G2 cells based on determinations of morphological changes, cell cycle, and apoptosis. Nonetheless, Silva et al. [30] investigated the effects of *A. arguta* fruit extracts, prepared as infusions and decoctions, on Caco-2 and HT29-MTX cell lines. They reported an absence of cytotoxic effects on both cell lines. More recently, Macedo et al. [19] explored the polyphenolic composition and bioactive properties of *A. arguta* fruit using ultrasound-assisted extraction and reported cytotoxic effects on human oral carcinoma cell lines, which aligns with the present study. However, in both previous studies, the cultivars used by the authors were not identified. Further studies are needed to draw conclusions about the potential anticarcinogenic effects of these cultivars.

## 3. Materials and Methods

### 3.1. Chemicals

Catechin, nitroetrazolium blue chloride (NBT), dihydrorhodamine (DHR) and sodium hypochlorite (NaOCl) were purchased from Sigma-Aldrich, Hamburg, Germany. Dimethylformamide (DMF), disodium (Na_2_HPO_4_) and monopotassium phosphate (KH_2_PO_4_) were obtained from Merck, Hamburg, Germany. β-nicotinamide adenine dinucleotide (NADH) and phenazine methosulfate (PMS) were purchased from Sigma-Aldrich, Bangalore, India, and St. Louis, Missouri, USA, respectively. For HPLC analysis, 40 phenolic compounds from Sigma-Aldrich, Hamburg, Germany and Extrasynhtese, Genay, France with a purity at least ≥ 90%, belonging to different families, namely phenolic acids: gallic acid, protocatechuic acid, neochlorogenic acid, caftaric acid, chlorogenic acid, 4-*O*-caffeyolquinic acid, vanillic acid, caffeic acid, syringic acid, *p*-coumaric acid, ferulic acid, sinapic acid, 3,5-di-*O*-caffeoylquinic acid, ellagic acid, 4,5-di-*O*-caffeoylquinic acid, and cinnamic acid; flavonoids: catechin, epicatechin, naringin, naringenin, quercetin-3-*O*-galactoside, quercetin-3-*O*-glucopyranoside, rutin, myricetin, quercitrin, kaempferol-3-*O*-glucoside, isorhamnetin-3-*O*-glucoside, kaempferol-3-*O*-rutinoside, isorhamnetin-3-*O*-rutinoside, quercetin, tiliroside, kaempferol, apigenin, and chrysin; stilbenoids: *trans*-polydatin, resveratrol and *trans*-ε viniferin; chalcones: phloridzin and phloretin; and others: caffeine, were used in this study. Methanol and formic acid were gradient grade and obtained from Merck (Darmstadt, Germany). Individual Caco-2 (clone type C2Bbe1) and HT29-MTX cell cultures were acquired from American Type Culture Collection (ATCC, Manassas, Virginia, USA) and offered from Dr. T. Lesuffleur (INSERMU178, Villejuif, France), respectively. Dulbecco’s modified Eagle’s medium (DMEM) with GlutaMAX-I, fetal bovine serum (FBS), streptomycin, penicillin and amphotericin B were obtained from Invitrogen Corporation (Life Technologies, S.A., Madrid, Spain). Triton X-100 was purchased from Sigma Chemical Co. (St. Louis, Missouri, USA), while dimethylsulfoxide (DMSO) was obtained from AppliChem (Darmstadt, Germany).

### 3.2. Fruit Samples

The study was conducted on one cultivar of *A. arguta* (‘Geneva’), one cultivar of *A. polygama* (‘Pomarancheva’), and three cultivars of *A. kolomikta* (‘Tallinn’, ‘Vitakola’, ‘Pozdni’). All plants from which fruits were collected grew in the experimental garden at Warsaw University of Life Sciences (SGGW), Warsaw, Poland, under the same climatic and soil conditions. Fruits were collected at the stage of consumption maturity when they were coloured typically for the species, had black seeds and began to soften. For each species/cultivar, 900 g of fruit was collected randomly from two vines of each cultivar, then mixed and divided into equal parts of 300 g each. The basic parameters of the fruits (weight and SSC) were measured on 30 well-developed fruit samples. The results are summarized in Table 6. Other fruits were frozen in liquid nitrogen immediately after harvest and then freeze-dried. Freeze-dried fruits were vacuum-sealed in foil packages and stored under refrigeration at 4 °C until the extracts were prepared.

### 3.3. Ultrasound-Assisted Extraction (UAE)

*Actinidia arguta* fruit extracts were obtained by UAE using an ultrasonic probe processor (Sonic Vibracell, model VCX50, Newtown, CT, USA) associated with probe tip No. 630-0219 with 13 mm diameter. The extraction conditions were carried out as described by Silva et al. [17] for the optimal extract, using water as solvent, a solid:liquid ratio of 10% (w/v) and an ultrasonic intensity of 30 W/m^2^ for 31.11 min. Afterward, the extracts were filtered through a Whatman n° 1 paper (Sigma-Aldrich, Hamburg, Germany) and frozen at −80 °C for subsequent lyophilization (Telstar, model Cryodos-80, Barcelona, Spain). Then, dried samples were stored at room temperature until further analysis.

### 3.4. Total Phenolic Content (TPC)

The total phenolic content (TPC) was measured spectrophotometrically using a BioTek Instruments, Synergy HT GENS5 (St. Louis, Missouri, USA), based on a complex redox reaction, according to the Folin–Ciocalteu procedure [31], with minor modifications [32]. Gallic acid was used as a standard for calibration (curve linearity range = 5–100 μg/mL; *R*^2^ > 0.991). The results were expressed as mg of gallic acid equivalent (GAE) per gram of dry weight (dw).

### 3.5. Identification and Quantification of Phytochemical Compounds

A Shimadzu HPLC system (Shimadzu Corporation, Kyoto, Japan) equipped with a photodiode array detector (PDA) and a Gemini C18 column (250 × 4.6 mm, 5 μm) from Phenomenex (Torrance, CA, USA) was used for analysis, as previously described by Moreira et al. [33]. Methanol (solvent A) and water (solvent B), both acidified with 0.1% formic acid, served as the mobile phase at a constant flow rate of 1.0 mL/min. The following gradient program was applied: 0–5 min: 20–24% A; 5–7 min: 24–25% A; 7–10 min: 25–26% A; 10–11 min: 26–26.5% A; 11–18 min: 26.5% A; 18–25 min: 26.5–30% A; 25–50 min: 30–45% A; 50–60 min: 45–50% A; 60–70 min: 50–55% A; 70–90 min: 55–70% A; 90–100 min: 70–100% A. A 5-min post-run at the initial conditions was performed to re-equilibrate the column before the next injection. To identify the individual phenolic compounds, their retention times and UV–Vis spectra were compared to those of pure standards. Quantification was carried out at 280, 320, or 360 nm, depending on the maximum absorption wavelength of the identified phenolic compounds. Results were expressed as mg of each phenolic compound per 100 g of extract on dw (mg/100 g dw).

### 3.6. Determination of In-Vitro Antioxidant/Antiradical Activities

#### 3.6.1. Ferric Reducing Antioxidant Power (FRAP)

The ferric ion reduction antioxidant capacity (FRAP) was calculated based on the reduction of a ferric 2,4,6-trypyridyl-s-triazine complex (Fe^2+^-TPTZ) to the ferrous form (Fe^3+^-TPTZ), as described by Benzie and Strain [34], with minor modifications. Ferrous sulfate 1 mM (FeSO_4_-7H_2_O) was used as standard (linearity range: 25–500 μM; *R*^2^ = 0.997). The results were presented as μmol of ferrous sulphate equivalent (FSE) per gram dw (μmol FSE/g dw).

#### 3.6.2. DPPH Radical Scavenging Assay

The DPPH radical scavenging assay was executed following the procedure described by Barros et al. [35], with minor modifications. The concentrations ranged between 31.25 and 1000 μg/mL. The results were presented in terms of IC_50_ (μg/mL).

### 3.7. Reactive Oxygen Species Scavenging Capacity

#### 3.7.1. Superoxide Radical Scavenging Assay

The superoxide radical (O_2_^•−^) scavenging capacity was determined spectrophotometrically, as described by Gomes et al. [36]. The results were expressed in IC_50_ (μg/mL) of the reduction of NBT to a purple-colored diformazan by reaction with O_2_.

#### 3.7.2. Hypochlorous Acid Scavenging Assay

The uptake capacity of hypochlorous acid (HOCl) was determined by monitoring the effect of extracts on the HOCl-induced oxidation of dihydrorhodamine (DHR) to rhodamine, according to Gomes et al. [36]. Results were expressed as the inhibition, in IC_50_ (μg/mL), of HOCl-induced DHR oxidation.

### 3.8. Cell Viability Assay

The cell viability assay was performed using intestinal cells (Caco-2 and HT29-MTX) to evaluate the cytotoxic potential of samples. Passage 9–15 of Caco-2 and passage 47–52 of HT29-MTX cells were used. The vital mitochondrial dye 3-(4,5-dimethylthiazol-2-yl)-2,5-diphenyltetrazolium bromide (MTT) assay was performed according to the methodology described by Pinto et al. [37]. Triton X-100 1% (w/v) and DMEM were used as negative and positive controls, respectively. Results were expressed as percentages (%) of cell viability.

### 3.9. Statistical Analysis

All assays were performed in triplicate and the results were presented as mean ± standard deviation (SD) (*n* = 3). One-way ANOVA test, followed by the HSD Tukey post–hoc test, was employed to analyze data through IBM SPSS Statistics 27.0 software (SPSS Inc., Chicago, IL, USA). Pearson’s correlation was employed to assess relationships between antioxidant/antiradical activity, TPC, and each particular group of polyphenols.

## 4. Conclusions

This study marks the first detailed analysis of bioactive compounds in various *Actinidia* species and cultivars, highlighting their health-promoting potential. In terms of polyphenol content, the results arranged the species in descending order as follows: *A. kolomikta* had the highest content, followed by *A. arguta* and *A. polygama*, with *A. kolomikta* surpassing the other two by approximately 9.4 and 13 times, respectively. The research identified 16 phenolic acids, 2 flavanols, 2 flavanones, 10 flavonols, 3 flavones, and 6 other compounds, with caffeine as the dominant compound. Catechin was a significant component in *A. kolomikta* and *A. arguta*, constituting 76.2–85.4% and 27.2% of total phenolics, respectively. In contrast, *A. polygama* displayed lower phenolic content, dominated by chlorogenic acid, 4-*O*-caffeoylquinic acid, and flavonols like quercetin-3-*O*-galactoside and myricetin. *A. kolomikta* showed the highest free radical scavenging activity, followed by *A. arguta* and *A. polygama*. Additionally, the cell viability decreased with increasing concentrations of *Actinidia* extracts, highlighting a possible anti-cancer effect of *A. kolomikta* cultivars ‘Vitakola’ and ‘Tallinn’ on Caco-2 cells, as reported for this species by other authors.

## Figures and Tables

**Table 1:** Total phenolic content (TPC) and in vitro antioxidant activities (evaluated by FRAP, DPPH, O_2_^•−^ and HOCl) of Actinidia extracts. Values are expressed as mean ± standard deviation (*n* = 3). Different superscript letters in the same column indicate significant differences between mean values (*p* < 0.05).

Fruit Sample	TPC (mg GAE/g extract dw)	FRAP (μmol FSE/g extract dw)	DPPH (IC_50_ (µg/mL)	ROS	
				O_2_^•−^	HOCl
				IC_50_ (μg/mL)	
*A. arguta* ‘Geneva’	11.70 ± 1.38 ^c^	144.53 ± 15.06 ^c^	NA ^1^	NA	51.81 ± 2.42 ^e^
*A. polygama* ‘Pomarancheva’	8.31 ± 0.77 ^c^	134.21 ± 7.08 ^c^	NA	NA	45.61 ± 1.31 ^d^
*A. kolomikta* ‘Tallinn’	75.59 ± 5.87 ^b^	576.24 ± 6.41 ^b^	393.33 ± 23.63 ^b^	103.73 ± 7.43 ^c^	6.37 ± 0.57 ^a,b,c^
*A. kolomikta* ‘Vitakola’	110.05 ± 8.83 ^a^	1049.30 ± 117.30 ^a^	260.22 ± 9.18 ^a^	47.37 ± 2.27 ^b^	11.04 ± 0.12 ^c^
*A. kolomikta* ‘Pozdni’	74.35 ± 6.20 ^b^	517.96 ± 16.56 ^b^	415.96 ± 10.16 ^b^	136.61 ± 13.51 ^c^	9.35 ± 0.83 ^b,c^
				Positive Controls	
Gallic acid				6.34 ± 0.21 ^a^	2.60 ± 0.05 ^a,b^
Catechin				18.01 ± 0.34 ^a,b^	0.20 ± 0.01 ^a^

^1^ NA: No activity determined up to the highest tested concentration (1000 μg/mL). FSE—ferrous sulphate equivalent.

**Table 2:** Identification and quantification of the phenolic compounds present in *Actinidia* extracts. Results expressed as mean ± standard deviation (mg of compound/100 g dw).

Compound	Retention time (min)	*A. arguta* ‘Geneva’ (mg/100g dw)	*A. polygama* ‘Pomarancheva’ (mg/100g dw)	*A. kolomikta* ‘Tallinn’ (mg/100g dw)	*A. kolomikta* ‘Vitakola’ (mg/100g dw)	*A. kolomikta* ‘Pozdni’ (mg/100g dw)
Phenolic acids						
Gallic acid	5.6	49.4 ± 2.5	16.2 ± 0.8	222.9 ± 11.1	166.2 ± 8.3	78.2 ± 3.9
Protocatechuic acid	9.9	177.8 ± 8.9	12.2 ± 0.6	114.4 ± 5.7	63.8 ± 3.2	64.9 ± 3.2
Neochlorogenic acid	10.2	164.1 ± 8.2	45.5 ± 2.3	109.2 ± 5.5	88.2 ± 4.4	40.8 ± 2.0
Vanillic acid	20.7	4.6 ± 0.2	53.7 ± 2.7	13.2 ± 0.7	9.0 ± 0.5	9.4 ± 0.5
Caffeic acid	21.2	ND	12.7 ± 0.6	17.3 ± 0.9	15.4 ± 0.8	8.5 ± 0.4
Syringic acid	22.3	ND	ND	0.5 ± 0.0	0.3 ± 0.0	ND
Caftaric acid	15.4	137.5 ± 6.9	38.9 ± 1.9	696.9 ± 34.8	385.9 ± 19.3	406.6 ± 20.3
Chlorogenic acid	17.9	9.5 ± 0.5	79.1 ± 4.0	65.3 ± 3.3	41.1 ± 2.1	47.0 ± 2.4
4-*O*-caffeyolquinic acid	19.9	17.7 ± 0.9	81.0 ± 4.0	143.8 ± 7.2	252.4 ± 12.6	171.2 ± 8.6
*p*-Coumaric acid	33.8	1.2 ± 0.1	6.6 ± 0.3	1.7 ± 0.1	0.5 ± 0.0	0.7 ± 0.0
Ferulic acid	37.3	<LOQ	13.3 ± 0.7	1.8 ± 0.1	3.5 ± 0.2	3.9 ± 0.2
Sinapic acid	37.7	ND	50.5 ± 2.5	2.0 ± 0.1	6.9 ± 0.3	6.5 ± 0.3
3.5-di-*O*-caffeoylquinic acid	50.1	8.9 ± 0.4	2.1 ± 0.1	ND	3.0 ± 0.2	1.0 ± 0.1
Ellagic acid	55.3	8.4 ± 0.4	42.8 ± 2.1	4.5 ± 0.2	3.6 ± 0.2	4.0 ± 0.2
4.5-di-*O*-caffeoylquinic acid	56.8	13.5 ± 0.7	66.0 ± 3.3	5.1 ± 0.3	3.5 ± 0.2	3.8 ± 0.2
Cinnamic acid	58.5	ND	ND	0.8 ± 0.0	0.2 ± 0.0	0.4 ± 0.0
∑Phenolic acids		592.7 ± 29.6	520.5 ± 26.0	1399.3 ± 70.0	1043.5 ± 52.2	846.9 ± 42.3
Flavanols						
Catechin	14.1	236.7 ± 11.8	15.3 ± 0.8	5040.5 ± 252.0	7001.2 ± 350.1	5049.8 ± 252.5
Epicatechin	23.3	ND	2.2 ± 0.1	36.9 ± 1.8	39.3 ± 2.0	33.3 ± 1.7
∑Flavanols		236.7 ± 11.8	17.6 ± 0.9	5077.4 ± 253.9	7040.5 ± 352.0	5083.1 ± 254.2
Flavanones						
Naringin	49.8	1.9 ± 0.1	10.1 ± 0.5	7.7 ± 0.4	4.4 ± 0.2	3.9 ± 0.2
Naringenin	68.1	ND	ND	ND	ND	ND
∑Flavanones		1.9 ± 0.1	10.1 ± 0.5	7.7 ± 0.4	4.4 ± 0.2	3.9 ± 0.2
Flavonols						
Quercetin-3-*O*-galactoside	52.2	3.1 ± 0.2	62.4 ± 3.1	<LOD	1.0 ± 0.0	0.8 ± 0.0
Rutin	53.3	ND	ND	ND	ND	ND
Myricetin	57.9	ND	22.1 ± 1.1	38.1 ± 1.9	4.8 ± 0.2	18.7 ± 0.9
Kaempferol-3-*O*-glucoside	59.5	ND	ND	ND	ND	ND
Kaempferol-3-*O*-rutinoside	60.3	ND	5.0 ± 0.2	ND	ND	ND
Quercetin	71.0	ND	ND	2.0 ± 0.1	2.6 ± 0.1	2.4 ± 0.1
Tiliroside	76.2	ND	ND	1.4 ± 0.1	2.2 ± 0.1	1.7 ± 0.1
Kaempferol	79.9	ND	ND	4.9 ± 0.2	1.8 ± 0.1	0.8 ± 0.0
Quercetin-3-*O*-glucopyranoside	52.7	ND	ND	<LOQ	ND	ND
Isorhamnetin-3-*O*-glucoside	60.0	ND	ND	ND	ND	ND
Isorhamnetin-3-*O*-rutinoside	60.3	ND	<LOQ	26.2 ± 1.3	35.8 ± 1.8	32.4 ± 1.6
∑Flavonols		3.1 ± 0.2	89.4 ± 4.5	72.6 ± 3.6	48.2 ± 2.4	56.8 ± 2.8
Flavones						
Apigenin	81.4	ND	ND	2.4 ± 0.1	1.2 ± 0.1	4.4 ± 0.2
Chrysin	90.8	ND	ND	ND	ND	0.3 ± 0.0
Quercitrin	59.1	ND	ND	ND	ND	ND
∑Flavones		0.0	0.0	2.4 ± 0.1	1.2 ± 0.1	4.7 ± 0.2
Others						
Phloridzin	54.4	6.0 ± 0.3	27.8 ± 1.4	ND	ND	ND
Phloretin	72.3	ND	ND	0.9 ± 0.0	1.2 ± 0.1	1.1 ± 0.1
Resveratrol	52.5	ND	5.3 ± 0.3	ND	ND	ND
*trans*-ε viniferin	69.2	ND	ND	ND	ND	2.2 ± 0.1
Caffeine	16.2	28.5 ± 1.4	14.7 ± 0.7	50.6 ± 2.5	63.3 ± 3.2	46.3 ± 2.3
*trans*-polydatin	39.2	2.9 ± 0.1	2.3 ± 0.1	<LOQ	<LOQ	<LOD
∑Others		37.3 ± 1.9	50.2 ± 2.5	51.5 ± 2.6	64.6 ± 3.2	49.7 ± 2.5
∑ All compounds		869.82	687.80	6610.90	8202.40	6045.10

ND: Not detected; LOQ: Limit of quantification; LOD: Limit of detection.

**Table 3:** Determination of coefficients (*R*^2^) between antioxidant/antiradical activity, TPC and the different phenolic classes.

	FRAP	DPPH	HOCl	O_2_^•−^	TPC
FRAP	X				
DPPH	0.41	X			
HOCl	0.64	0.93 **	X		
O_2_^•−^	0.19	0.93 **	0.75	X	
TPC	0.94 **	0.66	0.84 *	0.42	X
Phenolic acids	0.45	0.64	0.72	0.42	0.58
Flavanols	0.90 *	0.73	0.89 *	0.50	0.99 ***
Flavanones	0.03	0.01	0.00	0.01	0.03
Flavonols	0.00	0.07	0.11	0.06	0.01
Flavones	0.13	0.80 *	0.60	0.94 **	0.32
Other compounds	0.75	0.20	0.44	0.07	0.63
Sum of phenolics	0.88 *	0.75	0.91 *	0.51	0.99 ***

Significance: *** *p* < 0.001; ** *p* < 0.01; * *p* < 0.05.

**Table 4:** Effects of extract exposure on the viability of HT29-MTX cell line at different concentrations, as measured by MTT assay. Values are expressed as mean ± standard deviation (*n* = 3).

Sample	Concentration (μg/mL)				
	62.5	125	250	500	1000
*A. arguta* ‘Geneva’	86.06 ± 16.05 ^a^	72.52 ± 8.50 ^a^	73.61 ± 6.91 ^a^	72.20 ± 9.34 ^a^	83.34 ± 18.16 ^a^
*A. polygama* ‘Pomarancheva’	98.40 ± 13.23 ^a^	102.67 ± 4.83 ^a^	92.26± 16.64 ^a^	94.43± 12.74 ^a^	91.15 ± 14.94 ^a^
*A. kolomikta* ‘Tallinn’	100.69 ± 11.78 ^a^	58.28 ± 5.07 ^b^	54.17 ± 7.77 ^b^	54.70 ± 10.47 ^b^	22.87 ± 3.28 ^c^
*A. kolomikta* ‘Vitakola’	95.51 ± 10.22 ^a^	67.91 ± 12.64 ^b^	51.15 ± 11.28 ^c^	51.36 ± 7.64 ^c^	41.24 ± 8.71 ^c^
*A. kolomikta* ‘Pozdni’	80.89 ± 7.44 ^a^	79.20 ± 6.17 ^a^	81.76 ± 11.62 ^a^	66.23 ± 5.89 ^a^	82.48 ± 19.65 ^a^

Different superscript letters (a, b, c) in the same sample represent significant differences (*p* < 0.05) between different concentrations, according to Tukey’s HSD test.

**Table 5:** Effects of extract exposure on the viability of Caco-2 cell line at different concentrations, as measured by MTT assay. Values are expressed as mean ± standard deviation (*n* = 3).

Sample	Concentration (μg/mL)				
	62.5	125	250	500	1000
*A. arguta* ‘Geneva’	70.07 ± 7.34 ^a^	63.54 ± 10.65 ^a,b^	61.73 ± 9.62 ^a,b^	65.53 ± 8.92 ^a,b^	51.24 ± 6.10 ^b^
*A. polygama* ‘Pomarancheva’	100.74 ± 11.85 ^a^	78.55 ± 6.21 ^b^	81.70 ± 9.83 ^b^	75.83 ± 10.88 ^b^	58.55 ± 6.71 ^c^
*A. kolomikta* ‘Tallinn’	75.33 ± 10.30 ^a^	72.27 ± 28.44 ^a^	69.90 ± 9.38 ^a^	56.56 ± 14.27 ^a^	14.34 ± 2.46 ^b^
*A. kolomikta* ‘Vitakola’	81.13 ± 11.72 ^a^	50.01 ± 10.32 ^b^	55.47 ± 4.41 ^b^	45.94 ± 7.90 ^b^	41.66 ± 4.71 ^b^
*A. kolomikta* ‘Pozdni’	107.45 ± 6.48 ^a^	77.94 ± 14.06 ^b^	79.92 ± 11.35 ^b^	67.81 ± 10.03 ^b^	60.60 ± 13.05 ^b^

Different letters (a, b, c) in the same sample represent significant differences (*p* < 0.05) between different concentrations, according to Tukey’s HSD test.

**Table 6:** Basic parameters of the tested fruits at harvest. Results are expressed as mean ± standard deviation (*n* = 30).

Fruit Sample	Weight (g)	Soluble Solid Content (SSC, %)	Harvest Date
*A. arguta* ‘Geneva’	8.4 ± 2.3	17.4 ± 1.6	10 September 2021
*A. polygama* ‘Pomarancheva’	6.3 ± 1.5	14.2 ± 0.9	15 September 2021
*A. kolomikta* ‘Tallinn’	4.5 ± 0.6	12.3 ± 1.1	5 August 2021
*A. kolomikta* ‘Vitakola’	5.6 ± 0.4	13.5 ± 1.3	5 August 2021
*A. kolomikta* ‘Pozdni’	3.7 ± 0.2	11.0 ± 0.9	5 August 2021

## Data Availability

Data are contained within the article and Supplementary Materials.

## Supplementary Materials

The following supporting information can be downloaded at: https://www.mdpi.com/article/10.3390/molecules30020246/s1, Figure S1: An example of an HPLC chromatogram.
