# Supplementary material for: Biological and Health-Promoting Potential of Fruits from Three Cold-Hardy Actinidia Species
Source: Molecules. 2025 Jan 9;30(2):246. doi: 10.3390/molecules30020246 (PMC11767855; doi:10.3390/molecules30020246)

Figure S1. HPLC-PDA chromatograms monitored at 280 nm for (a) polyphenols standard mixture of 5 mg/L and (b) an example of the analysed sample *A. kolomikta* 'Vitakola'. Peak identification: (1) gallic acid, (2) protocatechuic acid, (3) neochlorogenic acid, (4) catechin, (5) caftaric acid, (6) caffeine, (7) chlorogenic acid, (8) 4-O-caffeyolquinic acid, (9) vanillic acid, (10) caffeic acid, (11) syringic acid, (12) epicatechin, (13) p-coumaric acid, (14) ferulic acid, (15) sinapic acid, (16) *trans*-polydatin, (17) naringin, (18) 3,5-di-O-caffeoylquinic acid, (19) quercetin-3-*O*-galactoside, (20) resveratrol, (21) quercetin-3-*O*-glucopyranoside, (22) rutin, (23) phloridzin, (24) ellagic acid, (25) 4,5-di-O-caffeoylquinic acid, (26) myricetin, (27) cinnamic acid, (28) quercitrin, (29) kaempferol-3-O-glucoside, (30) isorhamnetin-3-O-glucoside, (31) kaempferol-3-O-rutinoside, (32) isorhamnetin-3-O-rutinoside, (33) naringenin, (34) *trans*-ε viniferin, (35) quercetin, (36) phloretin, (37) tiliroside, (38) kaempferol, (39) apigenin, and (40) chrysin.

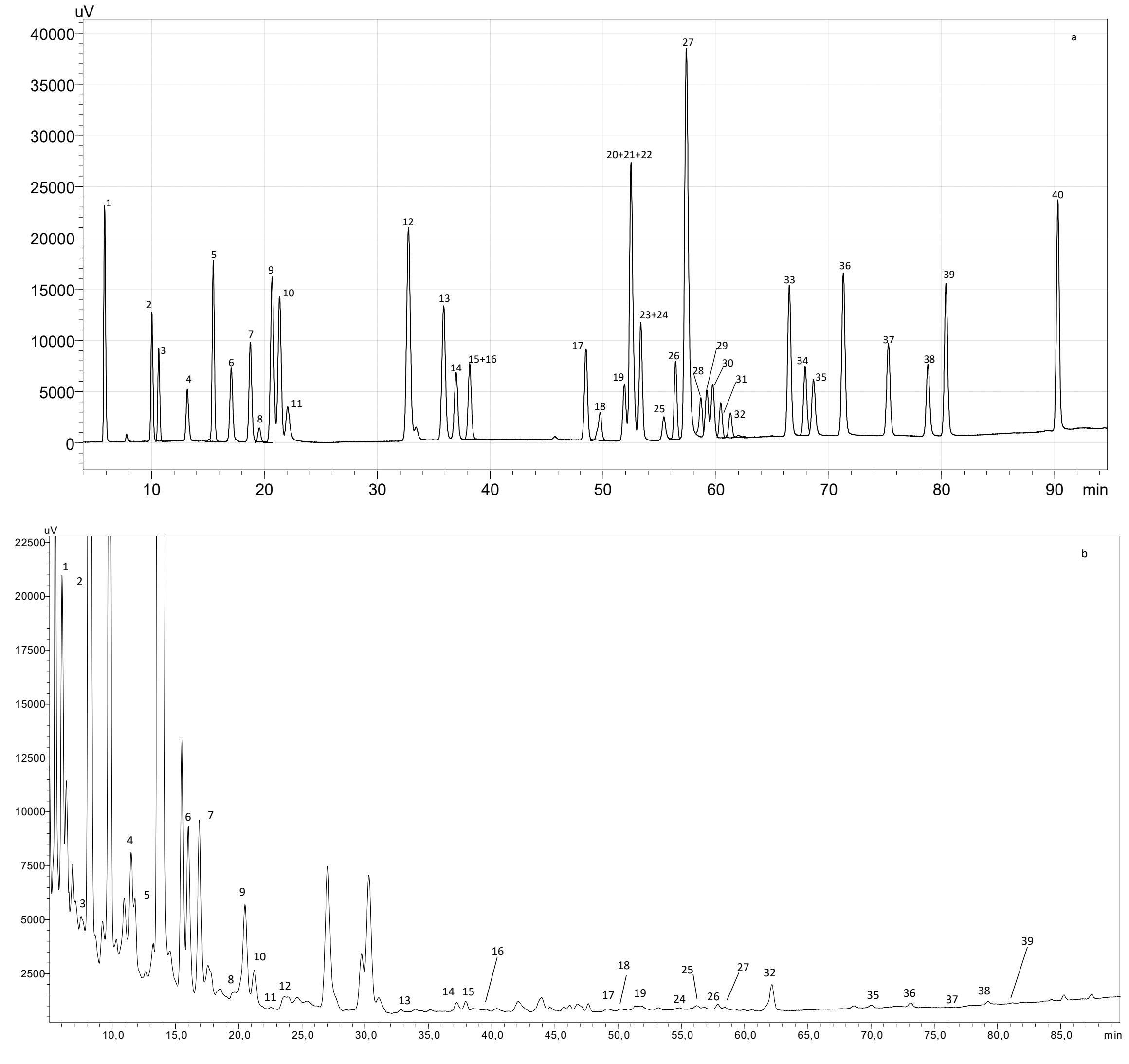

Supplement: Supplementary file 1 [file molecules-30-00246-s001.zip › molecules-3384075-supplementary.pdf]
